# Homotypic endothelial nanotubes induced by wheat germ agglutinin and thrombin

**DOI:** 10.1038/s41598-018-25853-3

**Published:** 2018-05-15

**Authors:** Lucia Pedicini, Katarina T. Miteva, Verity Hawley, Hannah J. Gaunt, Hollie L. Appleby, Richard M. Cubbon, Katarzyna Marszalek, Mark T. Kearney, David J. Beech, Lynn McKeown

**Affiliations:** Leeds Institute of Cardiovascular and Metabolic Medicine, School of Medicine, University of Leeds, Leeds, LS2 9JT UK

## Abstract

Endothelial barrier formation is maintained by intercellular communication through junctional proteins. The mechanisms involved in maintaining endothelial communication subsequent to barrier disruption remain unclear. It is known that low numbers of endothelial cells can be interconnected by homotypic actin-driven tunneling nanotubes (TNTs) which could be important for intercellular transfer of information in vascular physiology. Here we sought insight into the triggers for TNT formation. Wheat germ agglutinin, a C-type lectin and known label for TNTs, unexpectedly caused striking induction of TNTs. A succinylated derivative was by contrast inactive, suggesting mediation by a sialylated protein. Through siRNA-mediated knockdown we identified that this protein was likely to be CD31, an important sialylated membrane protein normally at endothelial cell junctions. We subsequently considered thrombin as a physiological inducer of endothelial TNTs because it reduces junctional contact. Thrombin reduced junctional contact, redistributed CD31 and induced TNTs, but its effect on TNTs was CD31-independent. Thrombin-induced TNTs nevertheless required PKCα, a known mediator of thrombin-dependent junctional remodelling, suggesting a necessity for junctional proteins in TNT formation. Indeed, TNT-inducing effects of wheat germ agglutinin and thrombin were both correlated with cortical actin rearrangement and similarly Ca^2+^-dependent, suggesting common underlying mechanisms. Once formed, Ca^2+^ signalling along TNTs was observed.

## Introduction

The endothelium is a regulated permeability barrier which determines exchange of proteins, fluids and immune cells in order to deliver appropriate inflammatory and haemostatic responses^1^. Chronic disruption of this barrier is characteristic of important disease states including oedema^2^, ischaemic and haemorrhagic stroke^3^, chronic inflammation^4,5^ and tumours^6,7^. Junctional proteins such as CD31 (PECAM-1) coordinate the opening and closing of inter-endothelial junctions and mediate cell-cell communication that is vital for the integrity of the vasculature under normal physiological conditions^8,9^, but how this communication is maintained when the endothelium is disrupted is poorly understood.

A putative mechanism of intercellular communication is Tunneling NanoTubes (TNTs) which are actin-driven extensions connecting remote cells and permitting transfer of diverse cellular components^10^. TNTs were first described in pheochromocytoma PC12 cells^11^ and have since been reported in rat cardiac myocytes^12^, human T cells^13^, epithelial cells^14^, rat hippocampal astrocytes^15^, MCF-7 human breast cancer cells^16^, chronic myeloid leukaemia cells^17^, murine bone-derived mast cells^18^ and human mesothelioma cells^19^. There are apparently low numbers of endothelial-to-endothelial homotypic TNTs with preference instead for heterotypic TNTs i.e. TNTs formed with other cell types^20^. Heterotypic endothelial TNTs have been suggested to mediate functions which include the rescue of premature senescence by progenitor cells^21^, the rescue of injured cells by stem cells^22,23^, the conferring of chemoresistance to mesenchymal cells^16^ and the transfer of miRNA from smooth muscle cells^24^. At present we have little understanding of the role of homotypic endothelial TNTs or how they are formed.

Further insight is required to appreciate the significance of endothelial TNTs in cardiovascular physiology and pathophysiology. Here we focused on homotypic endothelial TNTs to identify triggers and induction mechanisms which could point to contexts in which the TNTs become functionally important.

## Results

Human Umbilical Vein Endothelial Cells (HUVECs) have previously been shown to form low numbers of homotypic TNTs *in vitro*^21^. Here, we show that TNTs formed spontaneously between HUVECs in culture and, consistent with previous work, they were able to propagate mitochondria and plasma membrane (Supplementary Fig. S1A,B) suggesting that HUVECs are an appropriate model to explore endothelial TNT formation.

### Wheat germ agglutinin (WGA), a C-type lectin, induces TNTs

**Figure 1:**
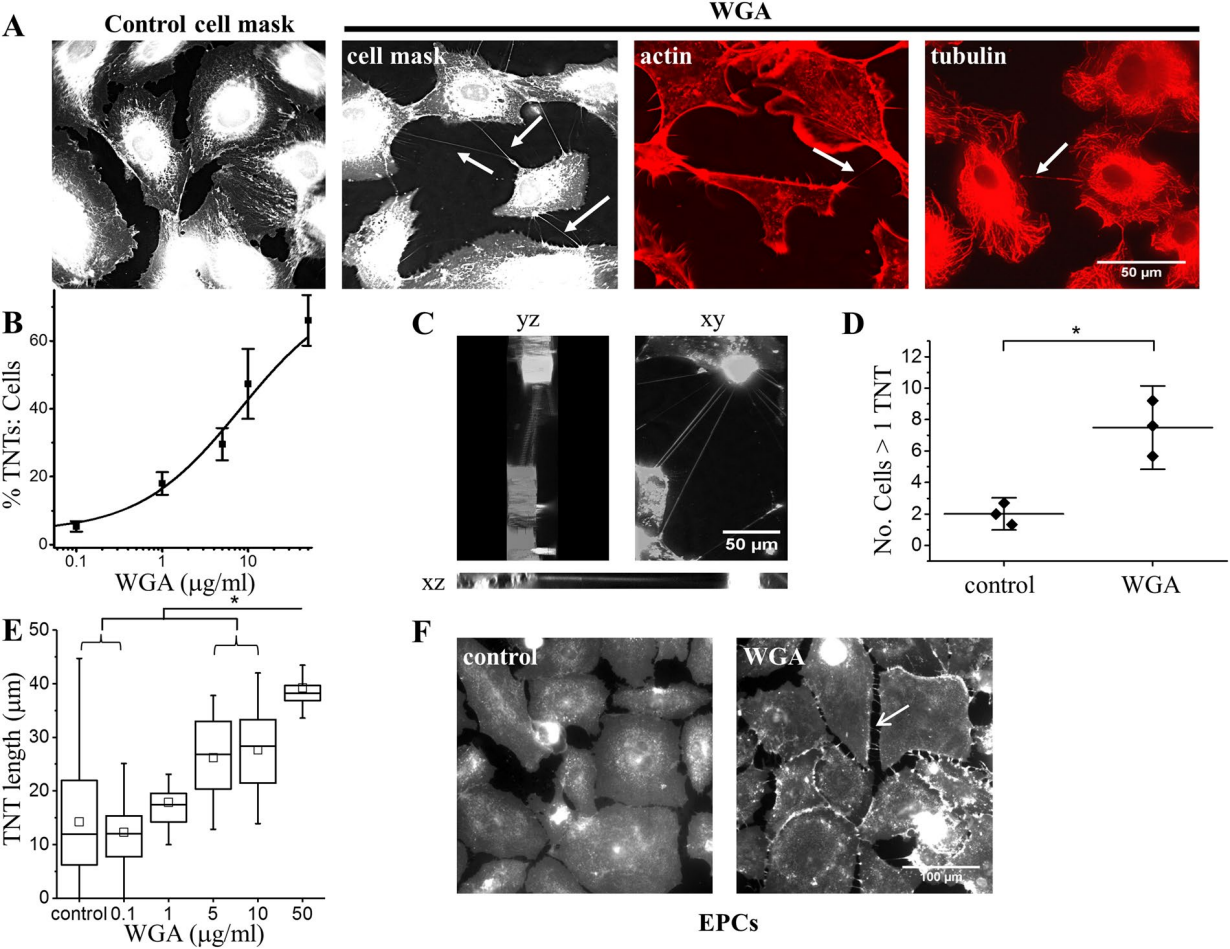
Wheat Germ Agglutinin (WGA) activates TNTs between endothelial cells. (**A**) Example images of HUVECs stained with cell mask (membrane), phalloidin (actin) or anti-tubulin (microtubules). Control cells display few or no TNTs. Incubation of HUVECs with 10 μg/ml WGA for 30 mins induced TNT formation (arrows) that contain actin (arrow) and/or microtubules (arrow). Scale bar 50 μm applies to all images. (**B**) Mean concentration response data and analysis from HUVECs treated with increasing amounts of WGA (n = 3 biological replicates for all groups, control N = 29, 0.1 N = 30, 1 N = 25, 5 N = 15, 10 N = 26. 50 N = 29) with a fitted Hill equation (EC_50_ 8.17 μg/ml). The data are the percentage of TNTs to cells. (**C)** DeltaVision captured images of WGA-induced TNTs connecting HUVECs taken as a stack of 36 × 0.5 μm slices. Yz and zx views depict the 3D volume of the cells shown in xy to illustrate TNTs hovering at different depths between the cells. Scale bar 50 μm. (**D**) Mean data depicting the significant increase number of cells treated with 10 μg/ml WGA for 30 mins that are connected by more than one TNT (i.e. forming a network) as compared to control (n = 3 for all groups, N = 29 control, N = 26 WGA). (**E**) Mean data and analysis of TNT length with increasing amounts of WGA. (n = 3 for all groups, N = 15–28 for each point). TNTs display varying lengths as indicated by the error bars but statistical analysis indicates a significant dose dependent increase in length (*). (**F**) Example images of endothelial progenitor cells stained with cell mask under control conditions or treated with 10 μg/ml WGA. Arrows depict TNTs. Scale bar is 100 μm and applies to both images. Data are represented as mean ± SD. *P < 0.05.

WGA is widely used to stain and observe TNTs in fixed and live cells^25^. Therefore we used WGA in our efforts to detect TNTs. Unexpectedly WGA induced the formation of TNTs (Fig. 1A–C). The effect was concentration-dependent with an EC_50_ of 8.17 μg/ml (Fig. 1B). WGA increased the number of inter-endothelial cell F-actin and tubulin containing connections that hovered above the substrate, consistent with identification of the structures as TNTs (Fig. 1A,C). The WGA-treated cells were able to form a network with multiple TNTs connecting cells over long distances (Fig. 1D,E). The effect also occurred in human endothelial progenitor cells (EPCs), so was not peculiar to HUVECs (Fig. 1F). The data suggest that WGA, a C-type lectin, induced TNT formation.

### Lectin subtype specificity and correlation with redistributed of F-actin

**Figure 2:**
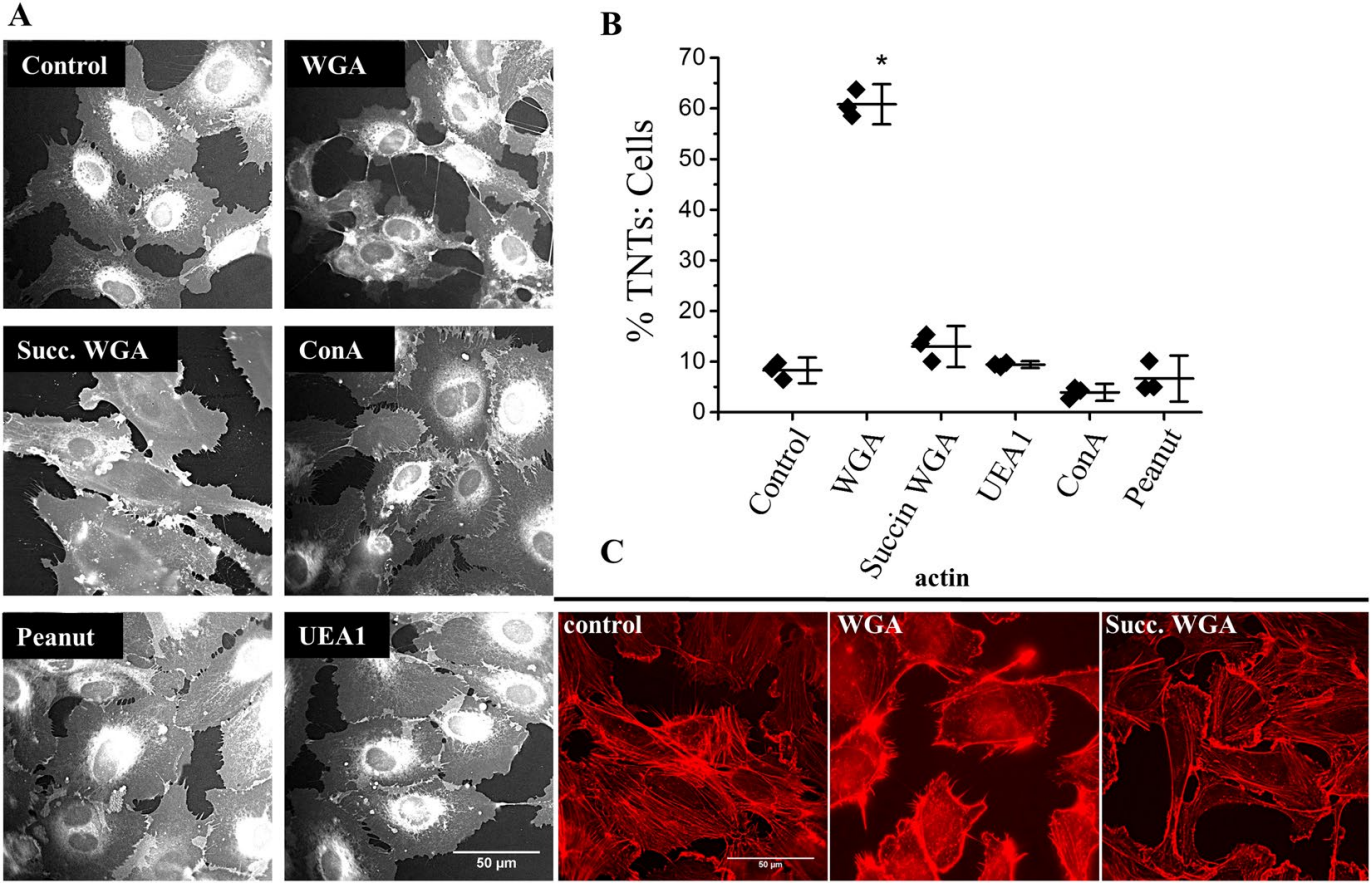
The sialic acid specificity of WGA induces endothelial TNT formation and F-actin rearrangements. (**A**) Examples of cell masked stained HUVECs following 30 mins incubation with the stated lectins (10 μg/ml). Scale bar is 50 μm. (**B**) Mean data and analysis of the effect of lectins on the percentage of TNTs to cells (n = 3, control N = 28, WGA N = 29, Succin WGA N = 30, UEA1 N = 30, ConA N = 29, peanut N = 30). Data are represented as mean ± SD, *P < 0.05. (**C**) Phalloidin staining (red) indicates F-actin redistribution in HUVECs treated with 10 μg/ml WGA for 30 mins as compared to control cells or cells treated with 10 μg/ml succinylated WGA. Scale bar is 50 μm.

Lectins bind to many carbohydrate molecules. To determine the specificity of the WGA effect we investigated other types of lectin, the binding properties of which are provided in Table S1. Only unmodified WGA induced TNT formation (Fig. 2A,B). Of particular note was succinylated WGA (a modified WGA containing a bulky succinyl group that binds N-acetylglucosamine but not to sialic acid^26^) which failed to induce TNTs. In marked contrast, WGA evoked F-actin distribution to a cortical arrangement whereas succinylated WGA did not (Fig. 2C). The data suggest that the induction of TNTs was specific to WGA and required binding to a sialylated protein.

### The WGA effect depends on CD31

**Figure 3:**
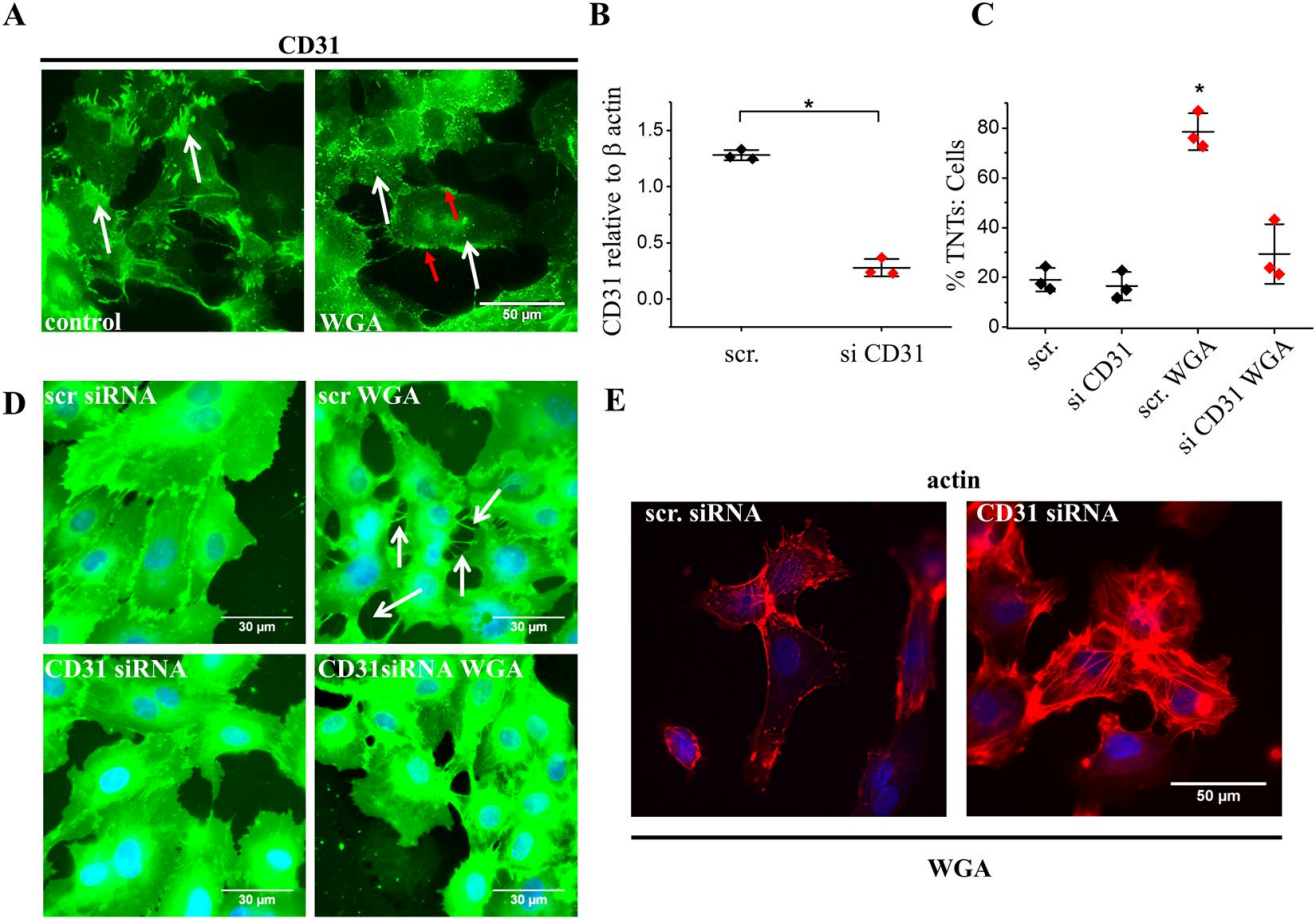
WGA mediated TNTs are dependent upon the junctional protein CD31. (**A**) Example images of HUVECs stained with a human anti-CD31 antibody (green) in control and cells treated with 10 μg/ml WGA for 30 mins. White arrows indicate the redistribution of CD31 from cell-cell contacts in control to clusters in WGA treated cells. Red arrows depict aggregates of CD31 at the plasma membrane Scale bar is 50 μm. (**B**) Mean data validating CD31 knock-down by RNA interference. In 3 independent experiments the CD31 siRNA reduced the protein abundance by ~87%. (**C**) Mean data and analysis (n/N = 3/29 each point) from images shown in D showing the effect of WGA is significantly inhibited when CD31 is reduced. Red diamonds indicate cells transfected with CD31 siRNA. Data are represented as mean ± SD. *P < 0.05. (**D**) Example images of cell mask stained cells depicting WGA-induced TNTs in HUVECS transfected with control siRNA (arrows) but not in cells transfected with siRNA targeted against CD31. Scale bar 30 μm. (**E)** Phalloidin staining (red) suggest that WGA does not induce F-actin redistribution in HUVECs transfected with CD31 siRNA as compared to control. Scale bar 50 μm.

We speculated that WGA might act via CD31 because CD31 is an important transmembrane protein of endothelial cells which is highly sialylated and previously suggested to be directly modulated by WGA^27^. We first tested if WGA affects the localisation of CD31 because binding of divalent antibodies directed against CD31 has been shown to induce protein clustering^28–30^. WGA caused redistribution of CD31 from cell-cell junctions to clusters across the cell surface (white arrows) and small aggregates at the plasma membrane (Fig. 3A: red arrows). Therefore, we depleted endothelial cells of CD31 using short interfering RNA (Fig. 3B). In CD31-depleted cells, WGA failed to induce significant TNT formation (Fig. 3C,D) or cortical redistribution of F-actin (Fig. 3E). The data suggest that the ability of WGA to induce TNTs depended on CD31. We postulated therefore that WGA evoked TNT formation by binding and clustering the sialic acid residues of CD31 thereby inducing cortical F-actin redistribution.

### Thrombin induces TNTs as well as F-actin and CD31 redistribution

**Figure 4:**
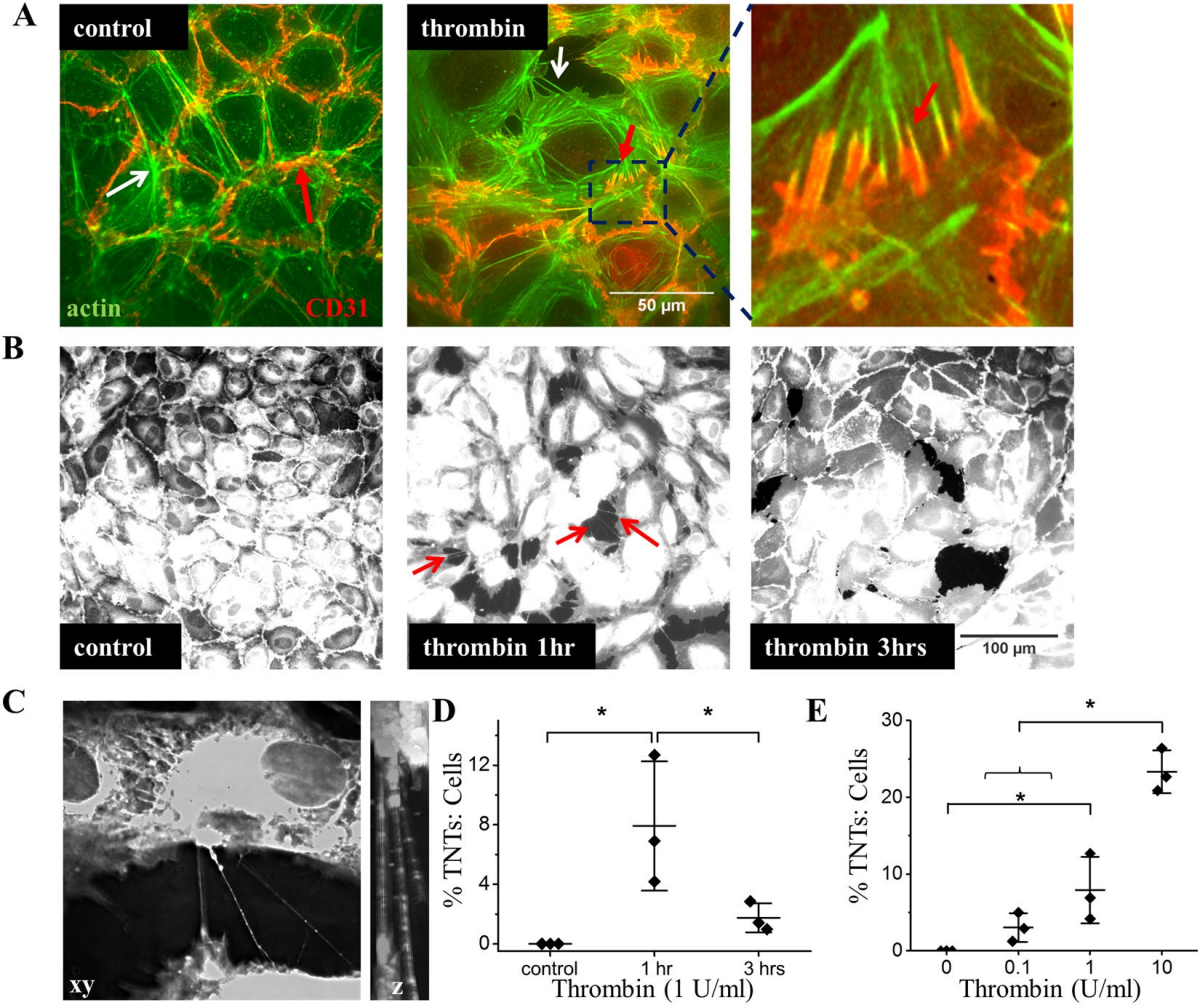
Thrombin induces TNTs as well as F-actin rearrangements and CD31 redistribution. (**A**) Example images showing actin (green) and CD31 (red) in HUVECs under control conditions or treated with 2 U/ml thrombin for 10 mins. Thrombin treated HUVECs display an accentuation of cortical actin. The white arrow depicts a TNT. The red arrows indicate the redistribution of CD31 in thrombin treated cells compare to control and the boxed area shows an enlargement of the CD31 staining (red) in the thrombin treated cells to further illustrate the arrangement of CD31 as compared to actin (green). Scale bar 50 μm. (**B)** Example images of cell mask stained cells showing the induction of TNTs (arrows) in HUVECs treated for 1 hr with 1 U/ml thrombin as compared to control cells or those treated for 3 hrs. Scale bar 100 μm. (**C)** DeltaVision captured images of thrombin-induced TNTs connecting HUVECs taken over 36 × 0.5 μm slices. The volume viewer depicts the hovering TNTs observed in the z plane. (**D**) Mean data from images shown in (**B**) showing significant changes in the number of TNTs formed between HUVECs treated with 1 U/ml of thrombin at the stated times (n = 3 biological repeats for each group. Control N = 15, 1 hr  N = 18, 3 hrs N = 15). (**E**) Mean data and analysis from HUVECs treated with increasing amounts of thrombin n = 3 biological repeats for each group. Control N = 15, 0.1U N = 16, 1U N = 17, 10U N = 13). The data are percentages of the number of TNTs to cells. Data are represented as mean ± SD. *P < 0.05.

We next sought to determine if the mechanism could be relevant to a physiological agonist. We focused on thrombin because it increases endothelial permeability and thus potentially creates fertile ground for TNT formation. We confirmed that thrombin increased inter-endothelial gaps in cells that were seeded at high density and grown to confluence in order to mimic the endothelial barrier (Supplementary Fig. S2). Application of thrombin to HUVECs led to accentuated cortical F-actin (Fig. 4A). Although CD31 did not cluster in response to thrombin, it changed distribution to follow the line of the cortical F-actin fibres (Fig. 4A, compare the area indicated by the red arrow in control as compared to the red arrow in the thrombin treated cells and the enhanced box area). Thrombin strikingly increased hovering TNT formation that lasted for about 1 hour and then declined (Fig. 4B–D) and the effect was concentration-dependent (Fig. 4E).

### Induction of TNTs by thrombin is CD31-independent

**Figure 5:**
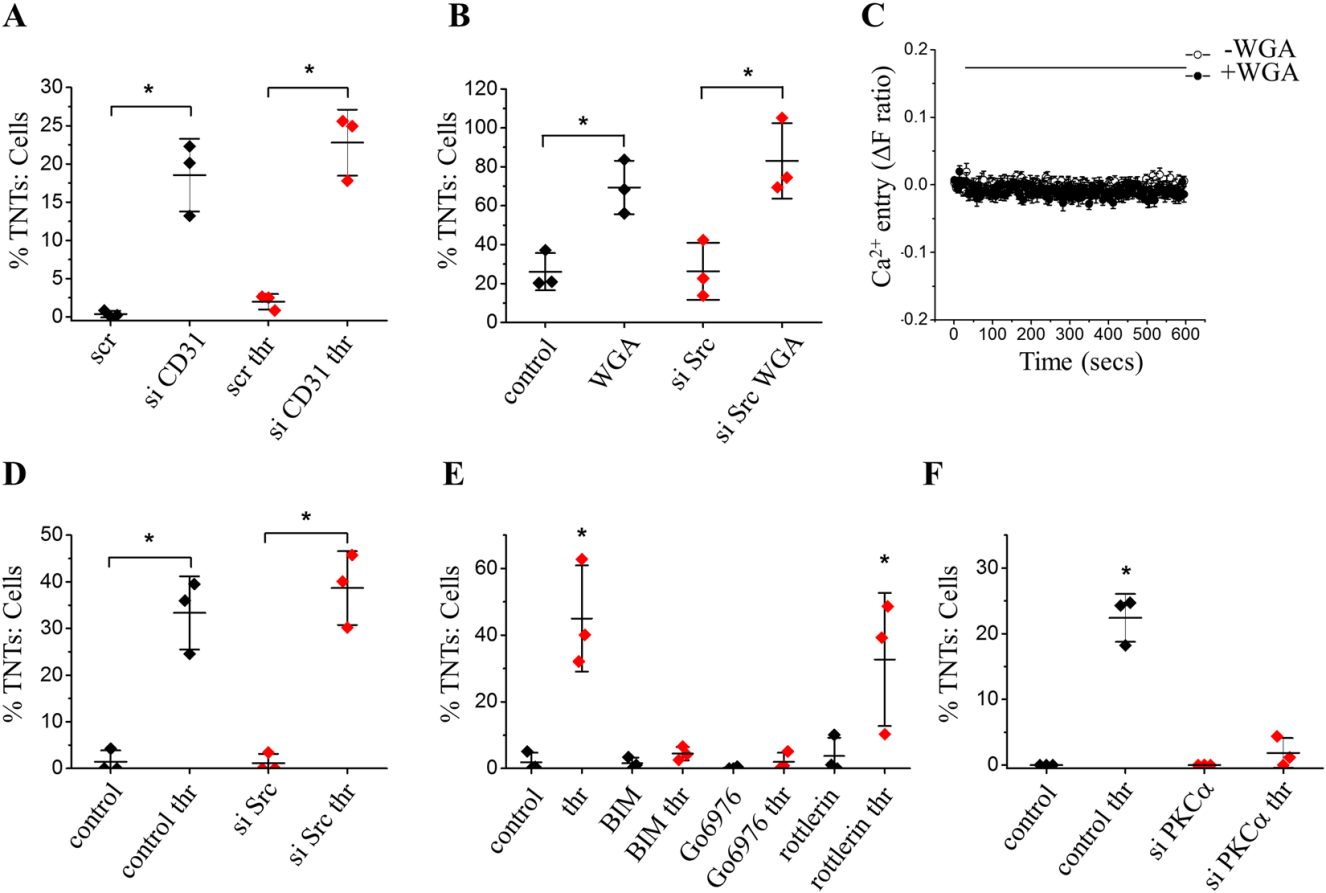
Thrombin-mediated  TNT induction is independent of CD31 but dependent on PKCα. (**A**) Mean data and analysis show that CD31 siRNA had no significant effect on the number of TNTs evoked by 2 U/ml thrombin (n/N = 3/17 for each group) as compared to siRNA control. (**B**) Mean data and analysis (n/N = 3/20 minimum for each group) from HUVECs transfected with siRNA specific to Src or a control siRNA +/− 10 μg/ml WGA showing WGA evoked significant increases in TNT formation in the presence or absence of Src kinase. (**C)** Example trace and mean data of HUVEC intracellular Ca^2+^ measurement in the presence of extracellular 1.5 mM Ca^2+^ showing responses to WGA (5 μg/ml) in Fura-2-loaded cells (n/N = 3/8 for each point). WGA failed to induce mobilisation of intracellular Ca^2+^. (**D)** Mean data and analysis (n/N = 3/23 minimum for each group) of HUVECs transfected with Src siRNA or siRNA control +/− 2 U/ml thrombin (thr). Thrombin significantly increased TNT formation in the presence or absence of Src kinase. (**E**) Quantification of TNT formation in cells pre-treated with the PKC inhibitor BIM, PKCδ blocker rottlerin or the PKCα selective inhibitor Go6976 prior to the addition of 2 U/ml thrombin (thr: n/N = 3/15–20 for each group). Significant formation of TNTs only occurred in those cells treated with thrombin alone or those pre-treated with rottlerin prior to thrombin treatment. (**F)** Mean data and analysis show that PKCα siRNA had a significant effect on the number of TNTs evoked by 2 U/ml thrombin (n = 3/N = 19 minimum for each group) as compared to siRNA control. All data are represented as mean ± SD. *P < 0.05.

To determine if CD31 is relevant to thrombin-induced TNTs we again depleted endothelial cells of CD31. In contrast to WGA, the thrombin effect was unaffected by CD31 depletion (Fig. 5A). Therefore, although there were similarities in the effects of WGA and thrombin, only the action of WGA depended on CD31.

### PKCα contributes to thrombin stimulated TNTs

In platelets, Ohmori *et al*. have shown that WGA activates the non-receptor tyrosine kinase Src and evokes rises in intracellular Ca^2+^ downstream of CD31 stimulation^27^. Here we show that in endothelial cells inhibition of Src by targeted siRNA (Supplementary Fig. S3A) had no effect on WGA induced TNT formation (Fig. 5B) and WGA failed to mobilise intracellular Ca^2+^ (Fig. 5C). We therefore propose that in endothelial cells WGA clamps cell-cell adhesion complexes via sialylated CD31, evoking TNT formation as the cells move apart. We therefore sought to understand the signalling mechanism by which the physiological activator, thrombin, induces TNTs. Thrombin acts through its proteolytically activated receptor 1 (PAR1) to evoke signalling events that impact upon the molecular organisation of endothelial adhesion complexes^31^. Both Src and the threonine/serine kinase PKC have roles in adherence junction remodelling^32–34^. Therefore, we explored the role of Src and PKC in thrombin stimulated TNT formation. HUVECs transfected with siRNA specifically targeted to Src kinase responded to thrombin (Fig. 5D) to a similar extent to cells transfected with control siRNA. These data suggest Src-mediated tyrosine phosphorylation does not play a role in TNT formation evoked by thrombin. To investigate the role of PKC we first used bisindolylmaleimide 1 (BIM), a selective total PKC inhibitor^35^. BIM treatment had no effect on its own but blocked thrombin-induced TNT formation (BIM; Fig. 5E). We investigated the role of PKC isoform specificity using rottlerin, a PKC δ inhibitor and Go6976, a PKCα inhibitor (rottlerin, Go6976; Fig. 5E). Pre-incubation with rottlerin had no effect on thrombin-induced TNTs but treatment of HUVECs with Go6976 significantly inhibited the effect of thrombin. To further test the role of PKCα we transfected HUVECs with PKCα siRNA (Supplementary Fig. S3B). Compared with a scrambled control siRNA, knockdown of PKCα significantly attenuated thrombin-induced TNT formation (Fig. 5F). The data indicates a significant role for PKCα on TNT formation upon thrombin exposure.

### WGA and thrombin induced TNT formation is Ca^2+^-dependent

**Figure 6:**
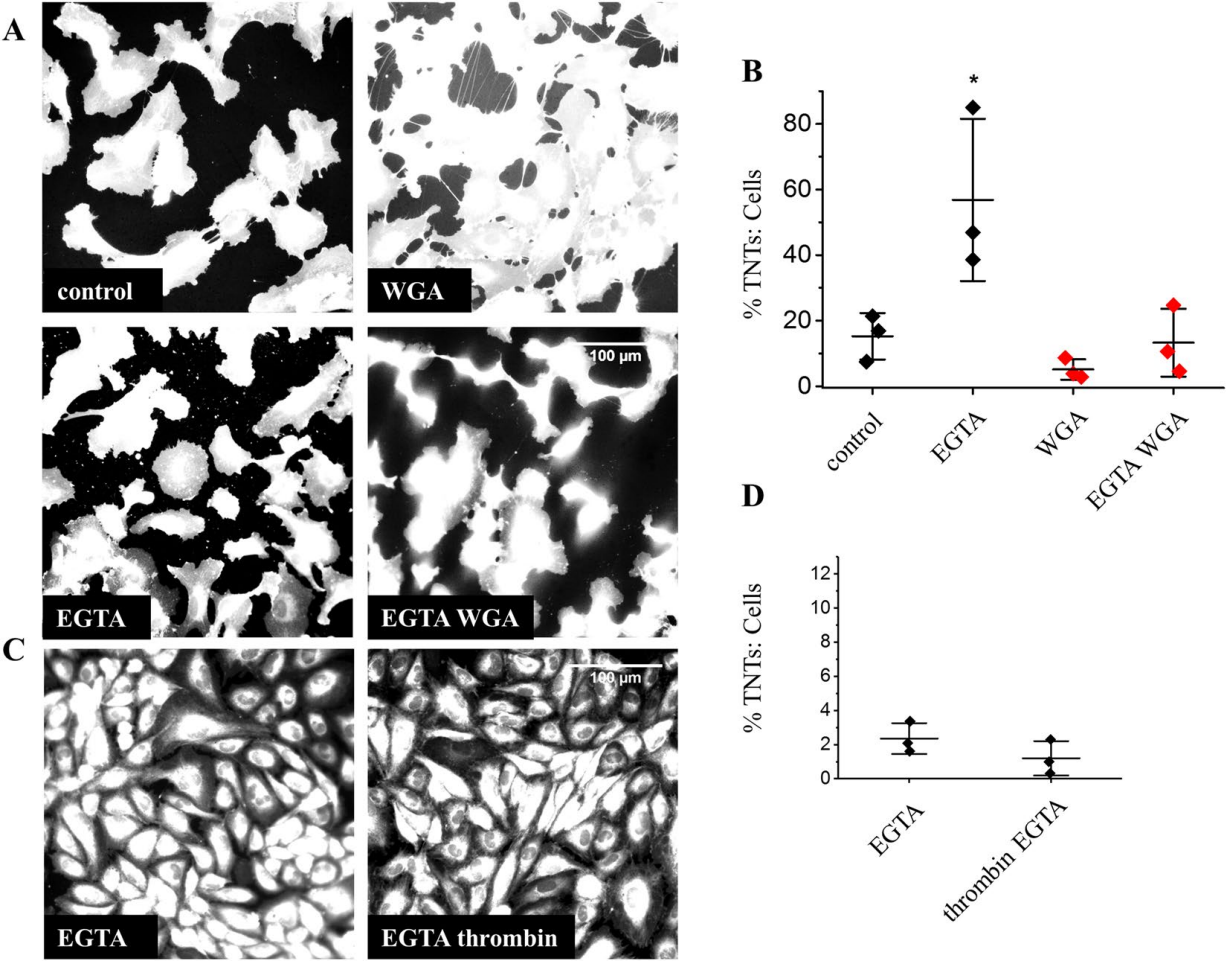
WGA and thrombin TNT formation is Ca^2^^+^-dependent. (A) Example images of cell mask stained HUVECs in control medium or medium with 5 mM EGTA +/− 10 μg/ml WGA showing a significant inhibition of WGA-induced TNTs in Ca^2+^ chelated medium. (**B**) Mean data (n/N = 3/30 for each group) of images in (**A**,**C)**. Example images of cell mask stained HUVECs in control medium containing 5 mM EGTA or medium with 5 mM EGTA + 1 U/ml thrombin. Thrombin did not evoke TNT formation in the presence of Ca^2+^ chelated medium (significantly different to the thrombin effect at 1 hr. in Fig. 4D). (**D)** Mean data (n/N = 3/15 for each group) of images in (**C**) and Fig. 4(B). Data are represented as mean ± SD. *P < 0.05. Scale bars are 100 μm.

Because of the similarities in the actions of WGA and thrombin on TNTs, we speculated that there might be a common downstream mechanism. We hypothesised that this might be a Ca^2+^-dependent process. Consistent with this suggestion, chelation of extracellular Ca^2+^ with EGTA mostly prevented the induction of TNT formation by WGA (Fig. 6A,B) or thrombin (Fig. 6C,D). The data suggest common requirement for extracellular Ca^2+^ in WGA and thrombin induction of TNTs.

### Homotypic endothelial TNTs support Ca^2+^ signalling

**Figure 7:**
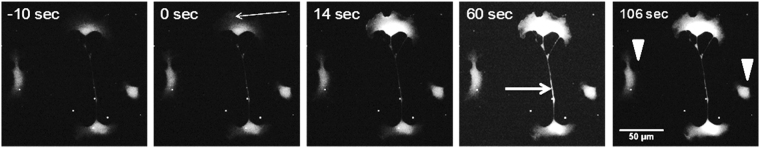
Homotypic endothelial TNTs mediate Ca^2+^ propagation. Fluo-4 loaded HUVECs were mechanically stimulated (time 0 secs: fine arrow) with a glass pipette. An increase in intracellular Ca^2+^ is shown by the change in fluorescence intensity in the stimulated cell (time 14 secs). Ca^2+^ is propagated along a TNT (60 secs: thick arrow) and into the connecting cell (time 106 secs). Unconnected cells (arrowheads) do not respond. Scale bar 50 μm.

We speculated that homotypic TNTs would mediate the transfer of ions in order to maintain barrier coordination. To test this suggestion we have observed the propagation of Ca^2+^ from a stimulated cell (Fig. 7: fine arrow at time 0: Movies 1 and 2) along a TNT (arrow at time 60 secs) to a connected cell (shown at 106 secs after stimulation). As observed in other studies^36,37^ Ca^2+^ propagated between a network of endothelial cells that were connected by TNTs or direct cell-cell contact (Supplementary Videos 1 and 2) but unconnected cells failed to respond (Fig. 7) unless there was cell damage (Supplementary Video 3). These data suggest that homotypic TNTs could maintain endothelial communication by the propagation of Ca^2+^ signals.

## Discussion

**Figure 8:**
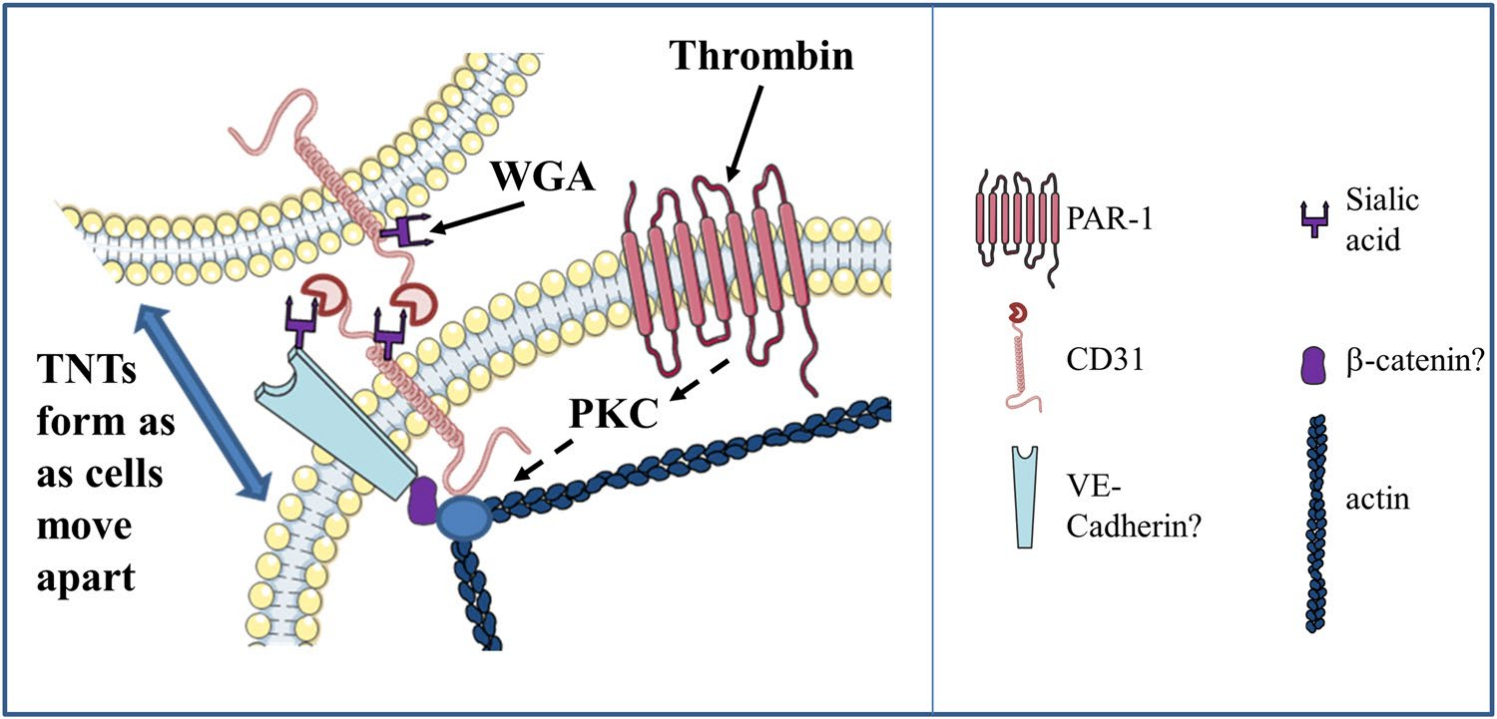
Schematic demonstration of TNT formation. WGA acts on sialic acid residues exposed on the extracellular surface of CD31 transducing signals to actin binding proteins and triggering changes in actin distribution. Thrombin acts via its PAR1 receptor initiating an intracellular signalling cascade involving PKCα and impacts upon actin binding proteins. TNTs are formed in endothelial cells as cells move apart.

Our findings suggest that endothelial cells maintain Ca^2+^ signals during an assault on barrier formation by forming intercellular TNTs. We provide important insight into the mechanisms that trigger endothelial-to-endothelial (homotypic) TNTs. We show striking ability of WGA to induce TNTs and reveal that this induction depends on the highly sialylated endothelial-to-endothelial contact protein CD31. CD31 dependence suggests that this type of induction is likely to be a relatively endothelial-specific phenomenon because CD31 expression is largely restricted to endothelial cells. As well as a potential approach to achieving relatively endothelial-specific TNTs, the CD31-dependent effect of WGA suggests an important relationship between TNT formation, mechanical force detection and the molecular mechanisms of cell-to-cell contact. CD31 is a lectin that displays sialic acid-dependent homophilic binding^29^, in addition to heterophilic lectin activity to other sialylated proteins^38^. In Fig. 8, we depict CD31 homotypically bound to sialic acid residues on the extracellular surface of juxtaposed cells. In addition, CD31 also interacts heterotypically with other sialylated junctional proteins such as VE-cadherin. Here, we postulate that WGA clusters sialic acid residues exposed on non-bound CD31 whilst aggregating an as yet unknown CD31-containing complex at the cellular junction. Thus, WGA anchors a sialic acid-dependent complex at cell-cell junctions permitting thin membrane extensions to form as cells move apart. The concept of regulated cell-to-cell contact being important in TNT induction is reinforced by our observation that thrombin-induced cell-separation readily led to the appearance of TNTs. Thrombin has previously been shown to act via proteolysis of PAR1 receptors on the endothelial membrane inducing an intracellular signalling cascade that impacts upon the cytoskeleton^31^. Although thrombin evoked TNT formation was CD31-independent, like CD31-dependent WGA-induced TNT formation this correlated with enhanced cortical F-actin, suggesting common downstream mechanisms. Indeed, Konstantoulaki *et al*. observed that thrombin induced aggregates of VE-cadherin and catenins (actin binding proteins) at the plasma membrane and, as cells retracted, thin membrane extensions were formed between the cells^34^. These remodelling events required PKC modifications of VE-Cadherin and β-catenin although the group had not characterised the intercellular connections as TNTs^39^. Here we extend these findings to show that thrombin signalling via PKCα leads to TNT formation. Future studies would explore this signalling pathway in order to understand the cell-cell junctional remodelling required for thrombin-induced TNT formation. The findings support the hypothesis that homotypic TNTs are relevant to endothelial biology and suggest contexts in which the TNTs might be induced in physiology and patho-physiology.

The mechanism of the Ca^2+^-dependence of TNT formation is unclear but a possibility is that it reflects Ca^2+^-dependence of VE-cadherin, a key Ca^2+^-dependent junctional protein of endothelial cells and modulator of actin organisation^40^. Junctional proteins are disassembled and endocytosed during extracellular Ca^2+^ depletion^41,42^ therefore the inhibition of TNT formation in Ca^2+^ free medium suggests they are necessary for TNT formation. WGA, but not succinylated WGA, failed to induce intracellular Ca^2+^ mobilization and inhibited VEGF-evoked Ca^2+^ elevation (Supplementary Fig. S4A,B), suggesting that the WGA effect on TNTs was not related to elevation of intracellular Ca^2+^. Similarly, although thrombin induces a dose-dependent Ca^2+^ response (Supplementary Fig. S4C), thrombin-mediated actin rearrangements have been suggested to be Ca^2+^-independent^43,44^.

CD31 is abundant in endothelial cells, a commonly used endothelial cell marker and a constituent of intercellular endothelial junctions where it tightens junctions and regulates leukocyte extravasation and other aspects of endothelial biology^8,45^. Desialylation by NEU1 sialidase plays a role in counteracting CD31’s positive impact on endothelial cell migration and angiogenesis^46^. In addition, evidence suggests CD31 plays a pivotal role in the rapid restoration of the vascular permeability barrier following inflammatory or thrombotic challenge^47^ (Supplementary Figure S2). As sialylation also plays important roles in the formation of junctional proteins further studies should elucidate the role of sialic acid in the induction of endothelial TNTs and their role in barrier restoration.

There have been few studies describing TNTs *in vivo* or *ex vivo*^48,49,12,50^, presumably because the technical challenges are substantial. Detailed studies of this biology, at least in mammalian vasculature, are likely to remain challenging until specific TNT tools are identified and developed for live *in vivo* imaging at high spatial resolution. Nevertheless, our findings suggest a framework for studies in which TNT formation is provoked by thrombin and modulated CD31. A potentially relevant modulator of CD31 is shear stress. CD31 has been suggested to act as a shear stress sensor in a triad or larger complex with VE-cadherin and VEGF receptor 2^51,52^. Dependence of the sensing of shear stress on the glycocalyx has been suggested and glycocalyx is a sialylated structure^53,54^. Perhaps when there is separation of endothelial cells there is desialylation that triggers TNT formation via CD31.

Although mitochondrial exchange between TNTs has been observed in cultured endothelial cells, this relatively slow propagation, which has been shown to have important functions in the rescue of distressed cells, is unlikely to be necessary for the preservation of intercellular communication during barrier disruption. Here we have shown that homotypic TNTs permit the propagation of Ca^2+^ to connecting cells. Whether this is dependent on Cx43-containing gap junctions and IP_3_ propagation as described in other studies^55^ requires further analysis. However, these data suggest that TNTs could maintain the Ca^2+^ oscillations and waves that are required for the spatiotemporal control of endothelial permeability.

WGA is a plant lectin widely used for staining plasma membranes and thus TNTs. Although CD31 is most abundant in endothelial cells, our observations suggest caution when using WGA as a label for TNTs even in non-endothelial cell types. There are reports of effects of WGA on cytoskeleton of enterocytes^56^ and granulocytes^57^. To further this research, WGA would serve as a useful tool to identify the exact molecular composition of the junctional complex required for TNT formation between endothelial cells. WGA is also a Ca^2+^-dependent (C-type) lectin. The human genome encodes for over 100 proteins that are classified as containing C-type lectin like domains (CLEC superfamily of lectins)^58^. Interestingly, there are members of this superfamily that play significant roles in the integrity or the function of the vasculature^59,60^. For instance, selectins are C-type lectins expressed on both immune cells and endothelial cells that are important for leukocyte migration across the endothelium^61^. It would be interesting to see whether these lectin-containing proteins induce TNT formation in order to preserve cell-cell signalling during endothelial barrier disruption.

In conclusion, this study strengthens the hypothesis that homotypic TNTs have under-appreciated importance in endothelial biology. Importantly it has identified molecular mechanisms by which the nanotubes can be regulated – specifically through modulation of sialylated CD31 and the action of thrombin via PKCα. Based on these findings we suggest that homotypic endothelial TNTs are likely to be important in vascular regions exposed to disturbed mechanical force, inflammation or thrombosis, in which contexts the TNTs may serve to facilitate endothelial cell coordination when gaps between endothelial cells emerge.

## Methods

### HUVEC culture

Human umbilical vein endothelial cells (HUVECs: Lonza) were maintained in endothelial growth medium (EGM-2) supplemented with 2% foetal calf serum (FCS) and growth factor bullet kit (Lonza). Experiments were performed on passage 2–5 cells. Cells were maintained at 37 °C in a 5% CO_2_ incubator.

### EPCs

Whole peripheral blood was drawn from subjects into EDTA coated tubes. All participants provided written informed consent, according to the declaration of Helsinki; ethical approval was provided by the Harrogate and Leeds (Central) research ethics committees. All procedures were carried out in accordance with these regulations. For EPC extraction an equal volume of Dulbecco Phosphate buffered saline (DPBS) was added to the EDTA tubes, and the resultant mixture was layered onto Ficoll paque plus. As per manufacturer’s instructions, density gradient centrifugation was carried out, and peripheral blood mononuclear cells (PBMCs) were aspirated from the buffy coat layer. These were washed and re-suspended with DPBS and subject to re-centrifugation at 300 × g for 10 mins at 18 °C. The cell pellet obtained was suspended in EGM-2 growth medium with EGM-2 single quot bullet kits and 10% foetal calf serum (FCS) (v/v), with an equivalent of 5 million cells per 2 ml of medium placed per well of a 6-well fibronectin coated plate. Cells were then incubated at 37 °C with 5% CO_2_. After 3 days, cells were washed with DPBS and fresh medium replaced. The cells firmly adherent between 4–7 days were predominantly early outgrowth EPCs. The phenotype of early EPCs was confirmed by staining with anti-CD31 using fluorescence microscopy.

### Short interfering siRNA

HUVECs were transfected at 90% confluence with 50 nM siRNA for CD31 (Applied Biosystems/Ambion), PKCα, Src (Dharmacon, Smartpool) or negative control using Lipofectamine 2000 in OptiMEM according to the manufacturer’s instructions (Thermofisher). 4–6 hrs later, transfection reagents were removed and 2 ml of EGM-2 medium added. Cells were used in experiments 48 hrs post transfection. Control siRNA was a 19-bp scrambled sequence with no significant homology to human gene sequences (Silencer Negative Control number 1: Ambion).

### Western Blot

Cells were harvested in lysis buffer containing 10 mM Tris, pH 7.5, 150 mM NaCl, 0.5 mM EDTA, 0.5% NP-40, Mini Complete protease inhibitors (Roche), and PhosSTOP phosphatase inhibitors (Roche). 20 µg protein was loaded on a 4–20% gradient gel and resolved by electrophoresis. Samples were transferred to PVDF membranes and labelled overnight with primary antibody: mouse anti-human β-actin (1:2000, Santa Cruz Biotechnology) or mouse anti-human CD31 (1:1000, Abcam). Species appropriate secondary antibodies and SuperSignal Femto detection reagent (Perbio Science) were used for visualisation.

### Immunofluorescence

Cells were detached (48-hour post transfection for siRNA experiments) and transferred to glass-bottomed 35 mm dishes, 30,000/dish (WGA) or 40,000/dish (thrombin), with fresh culture medium and allowed to spread for 24–48 hours. Cells were treated in serum-free M199 plus 10 mM HEPES medium for 30 mins then incubated with the stated concentration of lectin (Vector labs). Thrombin (Sigma) was used at 1–2 U/ml unless otherwise stated. One NIH U/ml of human thrombin activity is equivalent to 0.324 μg/ml (i.e. 9 nM). 5 mM EGTA was used in some experiments to chelate extracellular Ca^2+^. Lectin solution was replaced with fresh medium for live cell imaging or cells were fixed *in situ* with 1% glutaraldehyde/2% PFA for 10 mins to preserve the TNT structure. For pharmacological studies cells were serum starved for 30 mins prior to incubation for 30 mins with 5 µM bisindolylmaleimide 1 (BIM) or 5 µM rottlerin or 100 nM Go6976. Immunostained cells were permeabilised in 0.1% TritonX-100 for 10 mins at room temperature then incubated with anti-tubulin 1:500 (Covance) or anti-CD31 1:100 (Abcam) for 1 hr at room temperature, then washed and incubated with the appropriate secondary IgG (Stratech Scientific, Jacksons Immuno Research). Cells were stained with either Cell Mask (1 μl in 10 mls PBS), rhodamine phalloidin (Cytoskeleton Inc.), MitoTracker (Thermofisher) or WGA-FITC (Vector Labs) along with DAPI as per manufacturer’s instructions.

### Microscopy

Cells were visualized on an Olympus IX-70 inverted microscope using ×20, ×40 or ×60 UPLAN objectives supported by a DeltaVision deconvolution system (Applied Precision LLC) with SoftWorx image acquisition and analysis software. Images were captured on a Roper CoolSNAP HQ CCD camera at 0.05 secs exposure. Epifluorescence was recorded using filter sets for FITC/TRITC. To analyse hovering TNTs 35–40 0.5 μm z focal planes were captured and analysed with ImageJ orthogonal views, dynamic resplice or 3D project function.

For the analysis of WGA-induced TNTs random fields across the dish were captured in a blinded manner using the SoftWorx stage viewer. As thrombin-induced TNTs were more prevalent during gaps, to prevent bias imaging of gaps, a control dish was used to capture random fields of view where the points of image capture was marked onto the stage viewer and used as markers for all subsequent images. ImageJ was used post acquisition to process and prepare micrographs.

### Live cell Ca^2+^ imaging

A Zeiss Axiovet 200 microscope coupled to an Eppendorf FemtoJet microinjection system was used to capture intracellular Ca^2+^ changes in Fluo4-loaded HUVECs. A polished glass pipette mounted onto the microinjection system was used to stimulate the cells and both epifluorescence (FITC) and bright field images were simultaneously captured every 2 secs using OpenLab software. Images were further compiled using ImageJ software.

### Intracellular Ca^2+^ measurement

HUVECs were seeded in non-coated 96-well plates (NUNC) and incubated for 1 hr in 2 μM fura-2 AM or Fluo4-AM in standard bath solution (SBS) at 37 °C in the presence of 0.01% pluronic acid (Thermofisher). SBS contained (mM): NaCl 130, KCl 5, MgCl_2_ 1.2, CaCl_2_ 1.5, d-glucose 8, HEPES 10; Osmolality adjusted to 290 mOsm with NaCl; pH titrated to 7.4 with 4 M NaOH. Ca^2+^ free solution was SBS without added Ca^2+^. Cells were washed three times with SBS before measurements were made at room temperature (21 ± 2 °C) on a 96-well fluorescence plate reader (FlexStation II^384^, Molecular Devices). Fura-2 was excited at 340 and 380 nm and emission collected at 510 nm. Readings were made every 10 secs. The change (Δ) in intracellular Ca^2+^ concentration was indicated as the ratio of Fura-2 emission intensities for 340 nm and 380 nm excitation (Δ*F* ratio).

### Data analysis

Subsequent to confirming that TNTs hovered over the substrate, for consistency, TNTs were discerned if they connected 2 endothelial cells, were more than 5 μm in length and less than 1 μm in width. TNT length was analysed using the line and measurement function of ImageJ and TNTs counted using the cell counter. TNT counts are displayed as the percentage of TNTs to cells in a field of view. For thrombin-induced intercellular gap analysis images were segmented using the ImageJ threshold function, inverted and gaps analysed as particles measured as the percentage area of the field of view. Each time point was normalized to its own control but simplified to one control bar on the graph.

Data were analysed and figures prepared using Origin 8.0 software (OriginLab Corporation). 5–10 fields of view ×3 replicates were analysed for each condition and presented as n/N, which indicates the number of replicates (n) and the number of individual fields of view per point (N). Averaged data are shown as mean ± SD. Data were produced in pairs and analysed statistically with two-sample *t* tests or one-way ANOVA using OriginPro 8.6 software (OriginLab). In all cases statistically significant difference is indicated by *P < 0.05. All measurements performed blind to verify non-bias. FlexStation experiments data are presented as *n*/*N*, which indicates the number of independent experiments (*n*) and the number of individual wells (replicates) in the 96-well plate. Paired data sets were compared using two-tailed Student’s *t*-tests and expressed as mean ± standard deviation (SD) unless stated.

### Data Availability

All data generated or analysed during this study are included in this published article (and its Supplementary Information files).

## Electronic supplementary material

Supplementary Information

Video 1

Video 2

Video 3

## References

[1] Yuan, S. Y. & Rigor, R. R. *Regulation of Endothelial Barrier Functio*n. (2011 by Morgan & ClaypoolLife Sciences., 2010). 21634066

[2] Betz AL, Iannotti F, Hoff JT (1989). Brain edema: a classification based on blood-brain barrier integrity. Cerebrovascular and brain metabolism reviews.

[3] Fang W (2015). Attenuated Blood-Brain Barrier Dysfunction by XQ-1H Following Ischemic Stroke in Hyperlipidemic Rats. Molecular neurobiology.

[4] Flemming S (2015). Soluble VE-cadherin is involved in endothelial barrier breakdown in systemic inflammation and sepsis. Cardiovascular research.

[5] Muller-Redetzky HC, Suttorp N, Witzenrath M (2014). Dynamics of pulmonary endothelial barrier function in acute inflammation: mechanisms and therapeutic perspectives. Cell and tissue research.

[6] Lewis KM, Harford-Wright E, Vink R, Nimmo AJ, Ghabriel MN (2013). Walker 256 tumour cells increase substance P immunoreactivity locally and modify the properties of the blood-brain barrier during extravasation and brain invasion. Clinical & experimental metastasis.

[7] Papadopoulos MC, Saadoun S, Davies DC, Bell BA (2001). Emerging molecular mechanisms of brain tumour oedema. British journal of neurosurgery.

[8] Privratsky JR, Newman PJ (2014). PECAM-1: regulator of endothelial junctional integrity. Cell and tissue research.

[9] Dejana E (2004). Endothelial cell-cell junctions: happy together. Nature reviews. Molecular cell biology.

[10] Gerdes HH, Rustom A, Wang X (2013). Tunneling nanotubes, an emerging intercellular communication route in development. Mechanisms of development.

[11] Rustom A, Saffrich R, Markovic I, Walther P, Gerdes HH (2004). Nanotubular highways for intercellular organelle transport. Science.

[12] He K (2011). Long-distance intercellular connectivity between cardiomyocytes and cardiofibroblasts mediated by membrane nanotubes. Cardiovascular research.

[13] Sowinski S, Alakoskela JM, Jolly C, Davis DM (2011). Optimized methods for imaging membrane nanotubes between T cells and trafficking of HIV-1. Methods.

[14] Wittig D (2012). Multi-level communication of human retinal pigment epithelial cells via tunneling nanotubes. PLoS One.

[15] Wang Y, Cui J, Sun X, Zhang Y (2011). Tunneling-nanotube development in astrocytes depends on p53 activation. Cell death and differentiation.

[16] Pasquier J (2013). Preferential transfer of mitochondria from endothelial to cancer cells through tunneling nanotubes modulates chemoresistance. Journal of translational medicine.

[17] Mineo M (2012). Exosomes released by K562 chronic myeloid leukemia cells promote angiogenesis in a Src-dependent fashion. Angiogenesis.

[18] Fifadara NH, Beer F, Ono S, Ono SJ (2010). Interaction between activated chemokine receptor 1 and FcepsilonRI at membrane rafts promotes communication and F-actin-rich cytoneme extensions between mast cells. International immunology.

[19] Lou E (2012). Tunneling Nanotubes: A new paradigm for studying intercellular communication and therapeutics in cancer. Communicative & integrative biology.

[20] Ferrati S (2012). Inter-endothelial transport of microvectors using cellular shuttles and tunneling nanotubes. Small.

[21] Yasuda K (2011). Tunneling nanotubes mediate rescue of prematurely senescent endothelial cells by endothelial progenitors: exchange of lysosomal pool. Aging (Albany NY).

[22] Liu K (2014). Mesenchymal stem cells rescue injured endothelial cells in an *in vitro* ischemia-reperfusion model via tunneling nanotube like structure-mediated mitochondrial transfer. Microvascular research.

[23] Wang X (2016). Rescue of Brain Function Using Tunneling Nanotubes Between Neural Stem Cells and Brain Microvascular Endothelial Cells. Molecular neurobiology.

[24] Climent M (2015). TGFbeta Triggers miR-143/145 Transfer From Smooth Muscle Cells to Endothelial Cells, Thereby Modulating Vessel Stabilization. Circulation research.

[25] Benard M (2015). Structural and functional analysis of tunneling nanotubes (TnTs) using gCW STED and gconfocal approaches. Biology of the cell/under the auspices of the European Cell Biology Organization.

[26] Monsigny M (1979). Properties of succinylated wheat-germ agglutinin. European journal of biochemistry.

[27] Ohmori T (2001). Wheat germ agglutinin-induced platelet activation via platelet endothelial cell adhesion molecule−1: involvement of rapid phospholipase C gamma 2 activation by Src family kinases. Biochemistry.

[28] Elola MT, Blidner AG, Ferragut F, Bracalente C, Rabinovich GA (2015). Assembly, organization and regulation of cell-surface receptors by lectin-glycan complexes. The Biochemical journal.

[29] Kitazume S (2010). Alpha2,6-sialic acid on platelet endothelial cell adhesion molecule (PECAM) regulates its homophilic interactions and downstream antiapoptotic signaling. The Journal of biological chemistry.

[30] Zhao T, Newman PJ (2001). Integrin activation by regulated dimerization and oligomerization of platelet endothelial cell adhesion molecule (PECAM)-1 from within the cell. J Cell Biol.

[31] Klarenbach SW, Chipiuk A, Nelson RC, Hollenberg MD, Murray AG (2003). Differential actions of PAR2 and PAR1 in stimulating human endothelial cell exocytosis and permeability: the role of Rho-GTPases. Circulation research.

[32] Wang Y (2006). Integrins regulate VE-cadherin and catenins: dependence of this regulation on Src, but not on Ras. Proceedings of the National Academy of Sciences of the United States of America.

[33] Allingham MJ, van Buul JD, Burridge K (2007). ICAM-1-mediated, Src- and Pyk2-dependent vascular endothelial cadherin tyrosine phosphorylation is required for leukocyte transendothelial migration. Journal of immunology.

[34] Konstantoulaki M, Kouklis P, Malik AB (2003). Protein kinase C modifications of VE-cadherin, p120, and beta-catenin contribute to endothelial barrier dysregulation induced by thrombin. *American journal of physiology*. Lung cellular and molecular physiology.

[35] Komander D (2004). Interactions of LY333531 and other bisindolyl maleimide inhibitors with PDK1. Structure (London, England: 1993).

[36] Wang X, Bukoreshtliev NV, Gerdes HH (2012). Developing neurons form transient nanotubes facilitating electrical coupling and calcium signaling with distant astrocytes. PLoS One.

[37] Smith IF, Shuai J, Parker I (2011). Active generation and propagation of Ca2 + signals within tunneling membrane nanotubes. Biophysical journal.

[38] Wong CW (2000). PECAM-1/CD31 trans-homophilic binding at the intercellular junctions is independent of its cytoplasmic domain; evidence for heterophilic interaction with integrin alphavbeta3 in Cis. Mol Biol Cell.

[39] Kouklis P, Konstantoulaki M, Malik AB (2003). VE-cadherin-induced Cdc42 signaling regulates formation of membrane protrusions in endothelial cells. The Journal of biological chemistry.

[40] Bibert S (2008). Establishment of cell-cell junctions depends on the oligomeric states of VE-cadherin. Journal of biochemistry.

[41] Heimark RL, Degner M, Schwartz SM (1990). Identification of a Ca2(+)-dependent cell-cell adhesion molecule in endothelial cells. J Cell Biol.

[42] Kartenbeck J, Schmelz M, Franke WW, Geiger B (1991). Endocytosis of junctional cadherins in bovine kidney epithelial (MDBK) cells cultured in low Ca2 + ion medium. J Cell Biol.

[43] Thurston G, Turner D (1994). Thrombin-induced increase of F-actin in human umbilical vein endothelial cells. Microvascular research.

[44] Stolwijk JA (2016). Calcium Signaling Is Dispensable for Receptor Regulation of Endothelial Barrier Function. The Journal of biological chemistry.

[45] Mei H (2014). Regulation of endothelial cell barrier function by antibody-driven affinity modulation of platelet endothelial cell adhesion molecule-1 (PECAM-1). The Journal of biological chemistry.

[46] Lee C (2014). NEU1 sialidase regulates the sialylation state of CD31 and disrupts CD31-driven capillary-like tube formation in human lung microvascular endothelia. J Biol Chem.

[47] Lertkiatmongkol P, Liao D, Mei H, Hu Y, Newman PJ (2016). Endothelial functions of platelet/endothelial cell adhesion molecule-1 (CD31). Current opinion in hematology.

[48] Antanaviciute I (2014). Long-distance communication between laryngeal carcinoma cells. PLoS One.

[49] Ady JW (2014). Intercellular communication in malignant pleural mesothelioma: properties of tunneling nanotubes. Frontiers in physiology.

[50] Connor Y (2015). Physical nanoscale conduit-mediated communication between tumour cells and the endothelium modulates endothelial phenotype. Nat Commun.

[51] Coon BG (2015). Intramembrane binding of VE-cadherin to VEGFR2 and VEGFR3 assembles the endothelial mechanosensory complex. J Cell Biol.

[52] Conway DE (2013). Fluid shear stress on endothelial cells modulates mechanical tension across VE-cadherin and PECAM-1. Curr Biol.

[53] Vine AK (2009). Recent advances in haemostasis and thrombosis. Retina (Philadelphia, Pa.).

[54] Born GV, Palinski W (1985). Unusually high concentrations of sialic acids on the surface of vascular endothelia. Br J Exp Pathol.

[55] Lock, J. T., Parker, I. & Smith, I. F. Communication of Ca2 + signals via tunneling membrane nanotubes is mediated by transmission of inositol trisphosphate through gap junctions. *Cell Calcium*, 10.1016/j.ceca.2016.06.004 (2016). 10.1016/j.ceca.2016.06.004 PMC5035603 27388952

[56] Sjolander A, Magnusson KE (1988). Effects of wheat germ agglutinin on the cellular content of filamentous actin in Intestine 407 cells. European journal of cell biology.

[57] Ruggeri ZM, Ware J (1993). von Willebrand factor. FASEB journal: official publication of the Federation of American Societies for Experimental Biology.

[58] Zelensky AN, Gready JE (2005). The C-type lectin-like domain superfamily. The FEBS journal.

[59] Lee S (2017). Carbohydrate-binding protein CLEC14A regulates VEGFR-2- and VEGFR-3-dependent signals during angiogenesis and lymphangiogenesis. The Journal of clinical investigation.

[60] Lowe KL (2015). Podoplanin and CLEC-2 drive cerebrovascular patterning and integrity during development. Blood.

[61] McEver, R. P. Selectins: initiators of leucocyte adhesion and signalling at the vascular wall. *Cardiovascular research*, 10.1093/cvr/cvv154 (2015). 10.1093/cvr/cvv154 PMC4592324 25994174

