# Supplementary material for: Homotypic endothelial nanotubes induced by wheat germ agglutinin and thrombin
Source: Sci Rep. 2018 May 15;8:7569. doi: 10.1038/s41598-018-25853-3 (PMC5953990; doi:10.1038/s41598-018-25853-3)
Supplement: Supplementary file 1 — Supplementary Information [file 41598_2018_25853_MOESM1_ESM.docx]

**Homotypic endothelial nanotubes induced by wheat germ agglutinin and thrombin**

Lucia Pedicini, Katarina T Miteva, Verity Hawley, Hannah J Gaunt, Hollie L Appleby, Richard M Cubbon, Katarzyna Marszalek, Mark T Kearney, David J Beech and Lynn McKeown *

Leeds Institute of Cardiovascular and Metabolic Medicine, School of Medicine, University of Leeds, Leeds, LS2 9JT, UK.

**Supplementary Information**

**
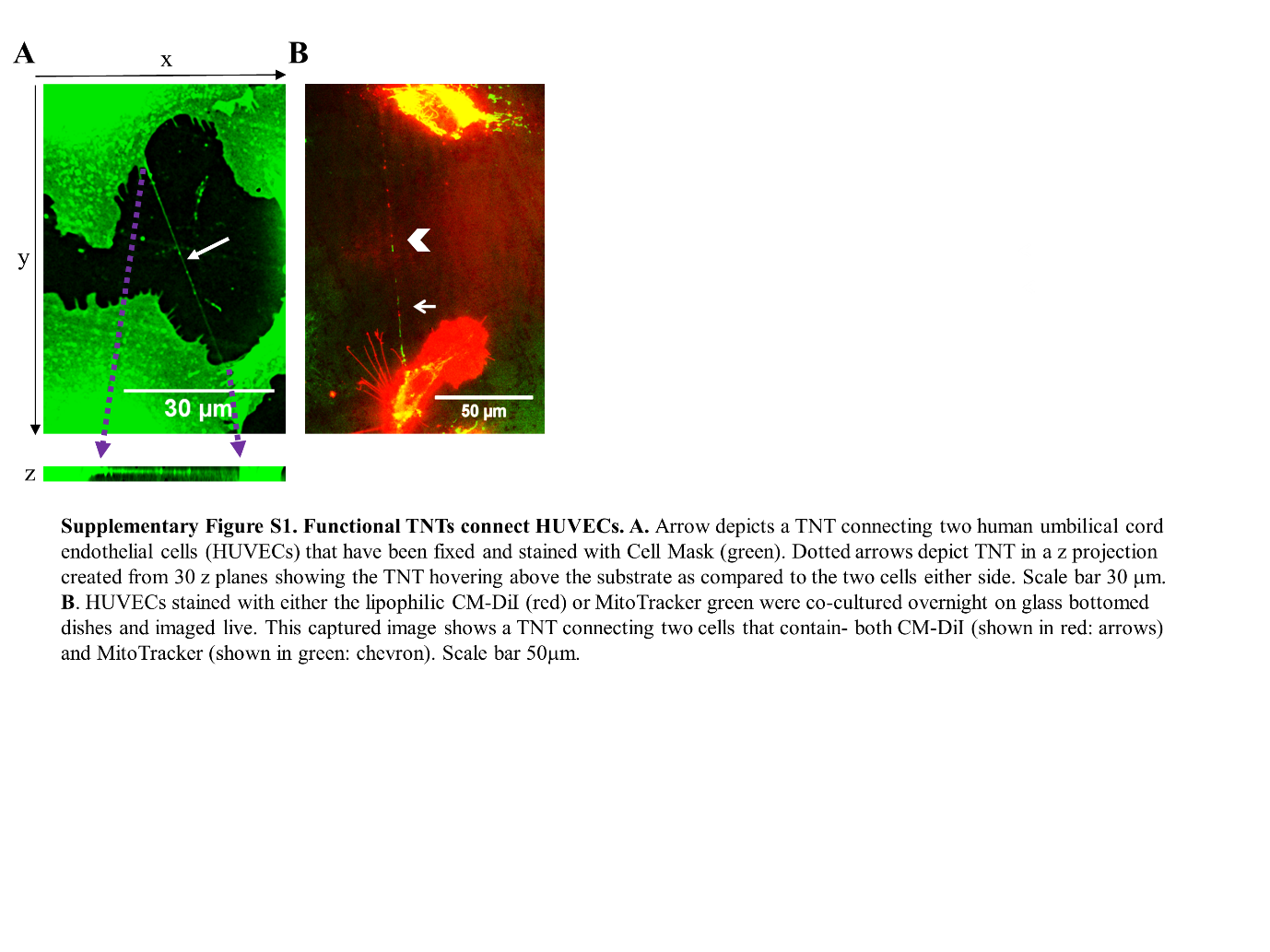
**

**
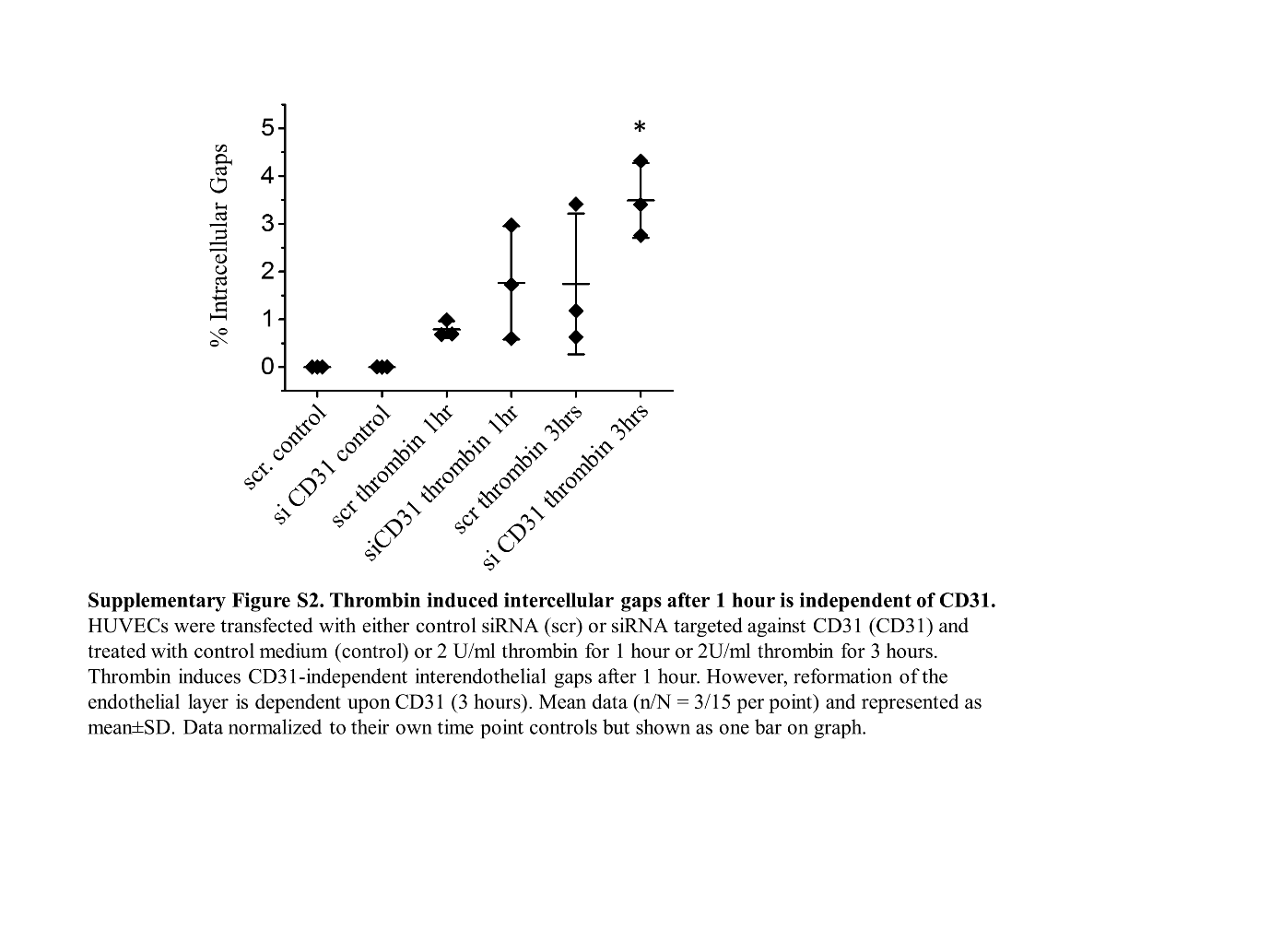

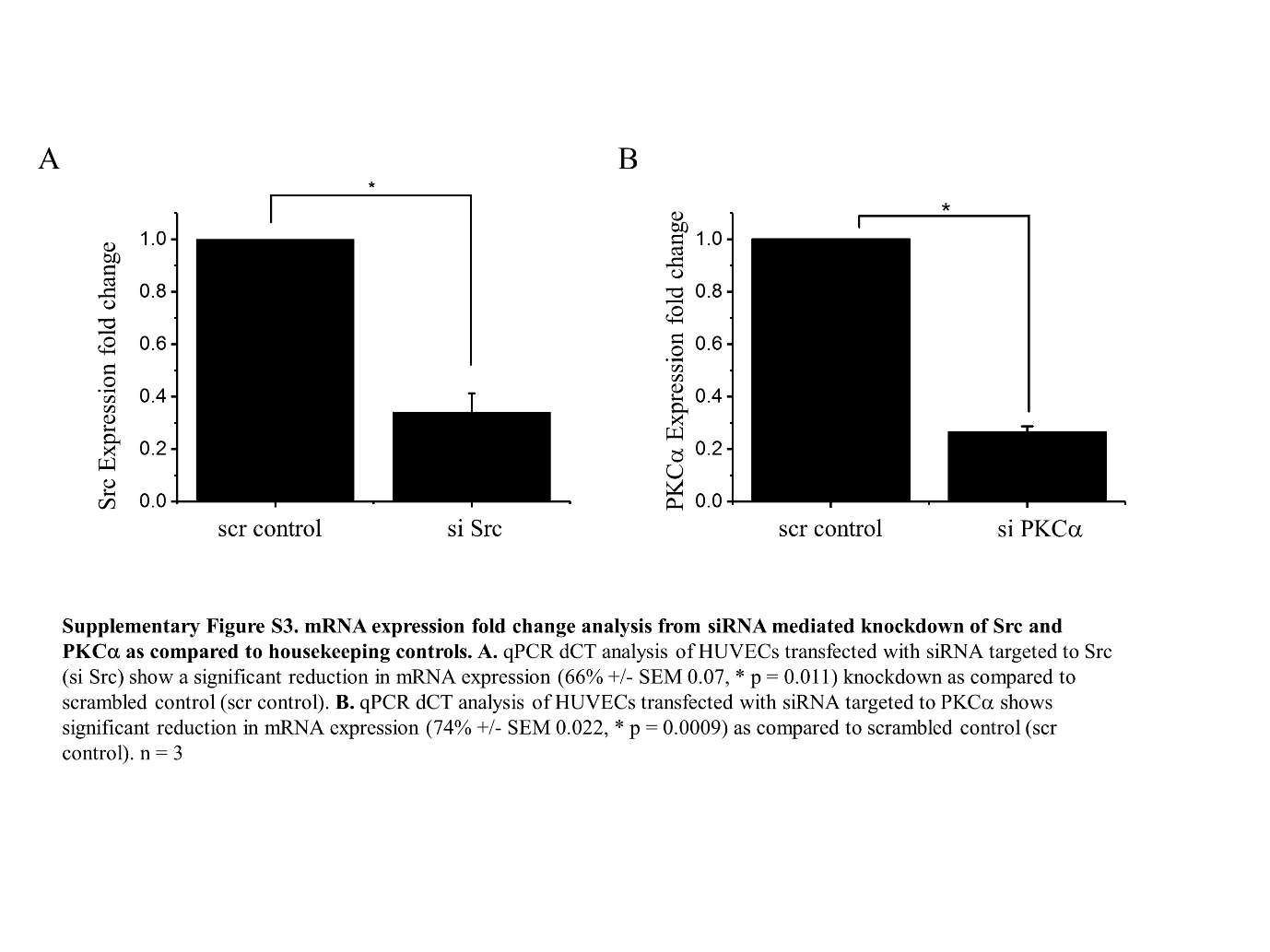

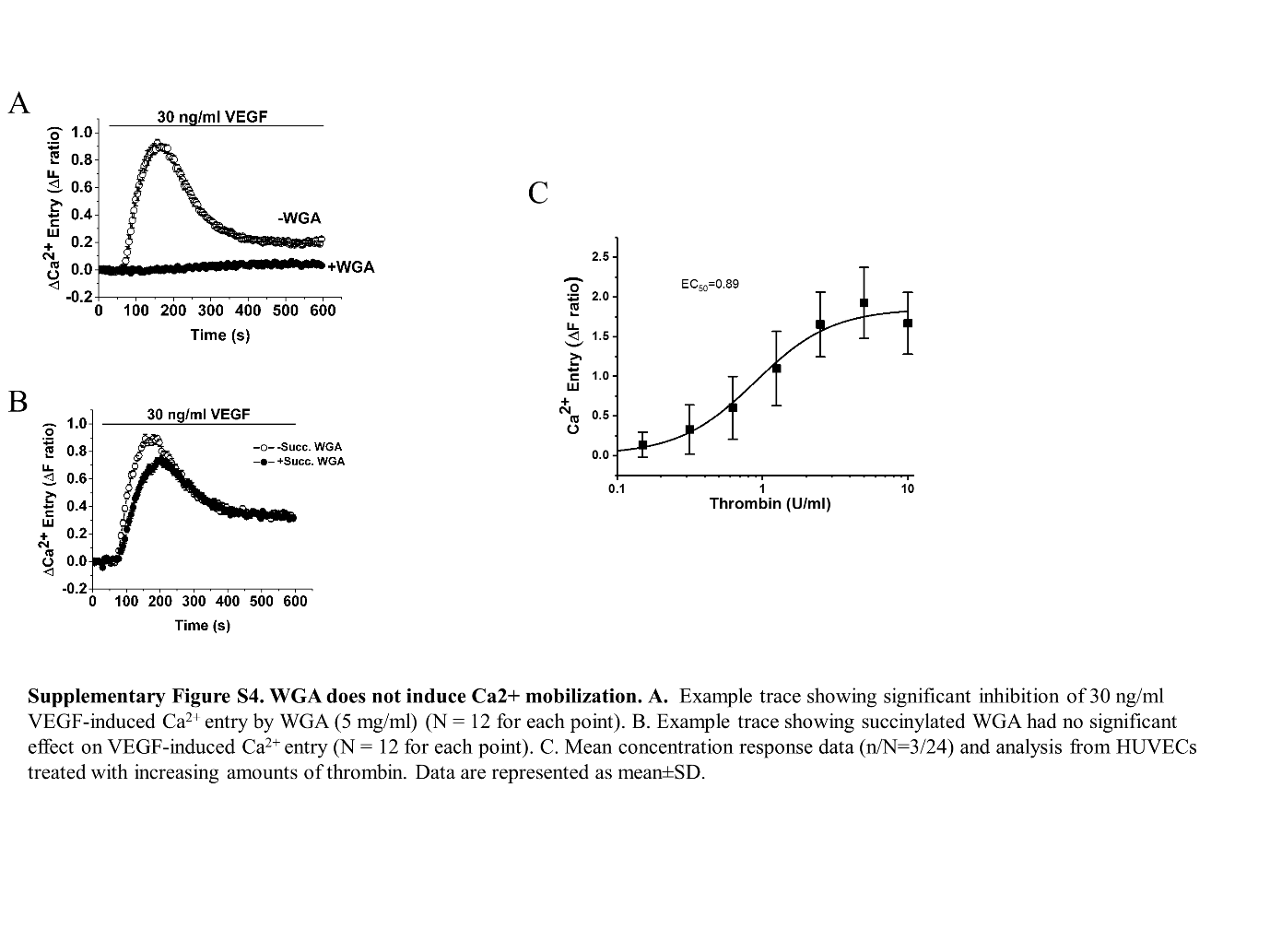

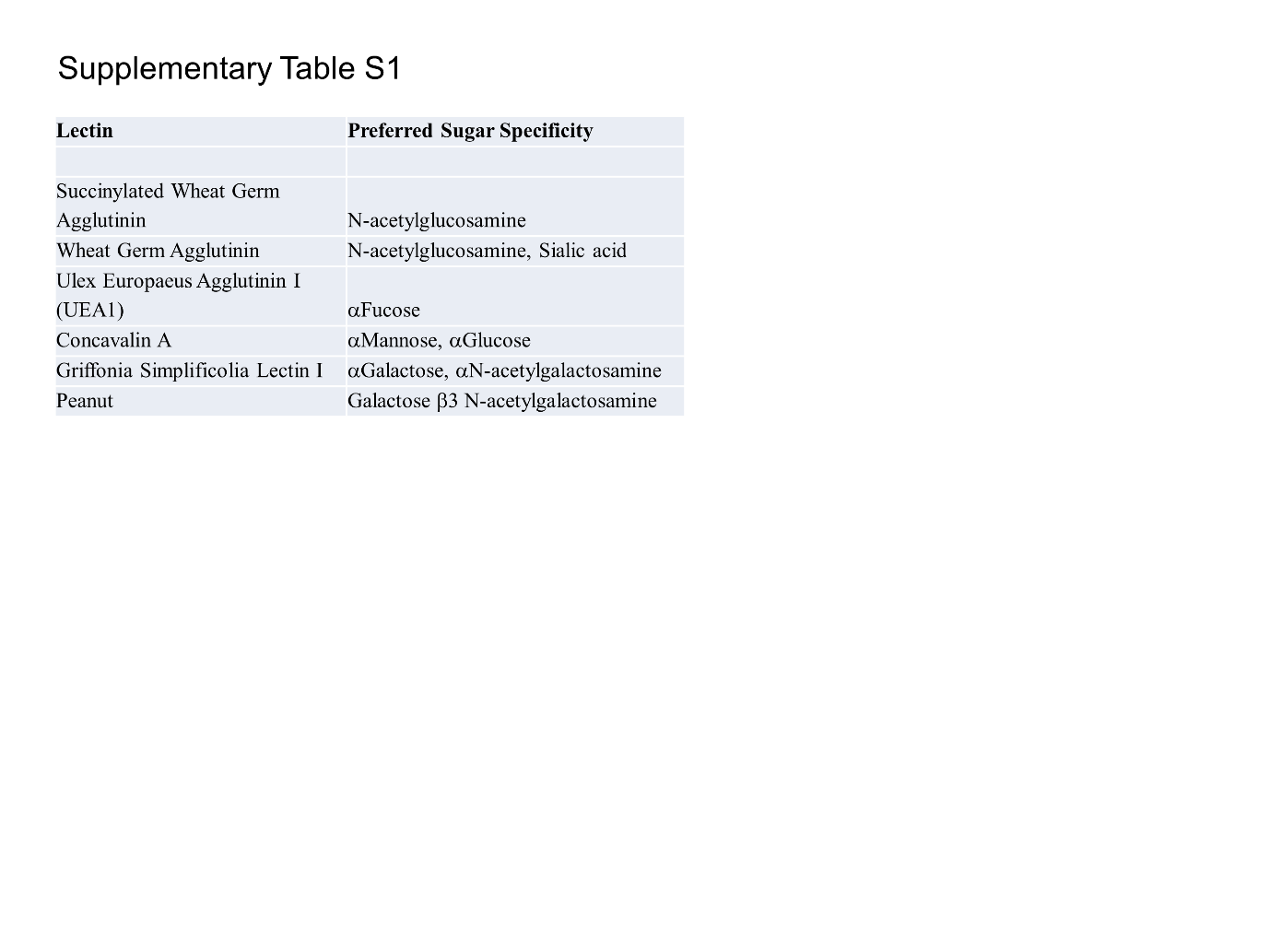

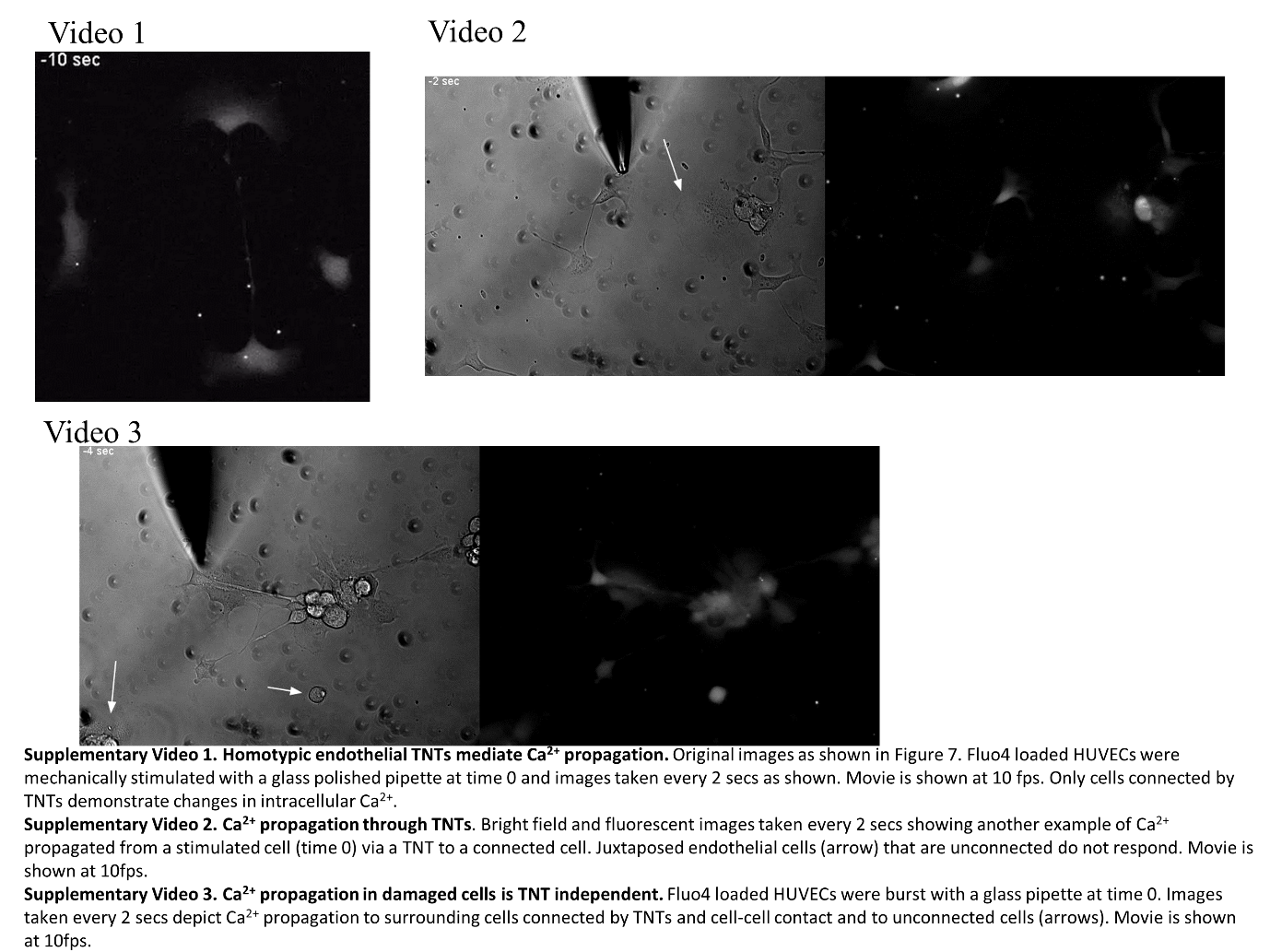
**
